# Physicochemical Characteristics of Protein Isolated from Thraustochytrid Oilcake

**DOI:** 10.3390/foods9060779

**Published:** 2020-06-11

**Authors:** Thi Linh Nham Tran, Ana F. Miranda, Aidyn Mouradov, Benu Adhikari

**Affiliations:** 1 School of Science, RMIT University, Bundoora Campus, Melbourne, VIC 3083, Australia; s3654084@student.rmit.edu.au (T.L.N.T.); ana.miranda@rmit.edu.au (A.F.M.); benu.adhikari@rmit.edu.au (B.A.); 2 Faculty of Agriculture Bac Lieu University, 8 wards, Bac Lieu 960000, Vietnam

**Keywords:** amino acids, emulsion, FTIR, protein secondary structure, proximate composition, surface hydrophobicity, thermal properties

## Abstract

The oil from thraustochytrids, unicellular heterotrophic marine protists, is increasingly used in the food and biotechnological industries as it is rich in omega-3 fatty acids, squalene and a broad spectrum of carotenoids. This study showed that the oilcake, a by-product of oil extraction, is equally valuable as it contained 38% protein/dry mass, and thraustochytrid protein isolate can be obtained with 92% protein content and recovered with 70% efficiency. The highest and lowest solubilities of proteins were observed at pH 12.0 and 4.0, respectively, the latter being its isoelectric point. Aspartic acid, glutamic acid, histidine, and arginine were the most abundant amino acids in proteins. The arginine-to-lysine ratio was higher than one, which is desired in heart-healthy foods. The denaturation temperature of proteins ranged from 167.8–174.5 °C, indicating its high thermal stability. Proteins also showed high emulsion activity (784.1 m^2^/g) and emulsion stability (209.9 min) indices. The extracted omega-3-rich oil melted in the range of 30–34.6 °C and remained stable up to 163–213 °C. This study shows that thraustochytrids are not only a valuable source of omega 3-, squalene- and carotenoid-containing oils, but are also rich in high-value protein with characteristics similar to those from oilseeds.

## 1. Introduction

The constant rise of the world’s population along with global climate change triggers strong demand for next generation sustainable and renewable feedstock for food and feed industries. This demand is driving increasing research into finding viable protein and protein-rich alternatives to crops and livestock. Currently, 67% of dietary protein is obtained from plants and 33% from animals, including fish [1]. Livestock is an expensive source of protein because of the decreasing availability of plant-based feed, clean water, and energy. Moreover, greenhouse gas emissions from livestock is another constrain of modern animal agriculture [1,2]. Production of plant proteins also has a limitation as it is restrained by decreasing arable land and freshwater, as well as climate change [1,3]. These challenges are becoming a driving force for the exploration of new cheap, renewable, and sustainable sources of protein that can be produced in climate-independent ways, such as in bioreactors. The seaweed and several species of microalgae (such as Spirulina) are now considered a viable source of protein containing an amino acid composition similar to those from meat, egg, soybean, and milk [4]. Plant-based oilcakes, the by-products of oil production industries from oilseeds, reported having protein contents in the range of 4–25% depending on the extraction process [5].

Protein concentrates and isolates are protein-enriched ingredients. While precise classification of concentrate and isolate in terms of protein content is still debated, concentrates contain <90% protein while isolates contain ≥90% on a dry mass basis. Commercially available protein concentrates contain 40–80% protein while isolates contain >90% protein on a dry solid basis. Both concentrates and isolates, especially milk protein concentrates and isolates, are widely used as ingredients in many food formulations because of their high nutritional value, as well as their unique techno-functional properties including water binding, emulsifying, gelling, and foaming [6]. Similarly, plant protein concentrates and isolates have also been increasingly produced and used over the last decade due to the popularity of plant proteins [7,8,9].

Thraustochytrids, marine unicellular, heterotrophic protists, are abundant in marine habitats of tropical, temperate and Antarctic waters [10,11,12]. They are important components of mangrove forest microbial ecosystems, recycling marine nutrients into a broad variety of biomolecules used as nutrients for both marine and terrestrial species. Thraustochytrids have been extensively studied over the last decade because of their biotechnological applications in human health based on their ability to produce very long chains of polyunsaturated fatty acids (VLC-PUFAs), mainly ω3-docosahexaenoic acid (DHA, C22:6) [13,14,15]. Health benefits of DHA include anti-inflammatory properties, cardiovascular protection, proper development of neural tissues and decreased risks of depression, Alzheimer’s, and Parkinson’s diseases [16,17,18]. The biomass from thraustochytrid species, which also contains carotenoids, proteins, and squalene, have been designated as Generally Recognized as Safe by the U.S. Food and Drug Administration (FDA) [19]. Thraustochytrids can also produce up to 38% dry weight (dw) of protein [15], which is lower in comparison to some common microalgae, such as *Spirulina* sp. (50–70% protein/dw) or *Chlorella* sp. (44.3% protein/dw) [20,21]. However, heterotrophic growth of thraustochytrids on a wide range of carbon sources showed much higher biomass productivity in a shorter period, making their protein productivity comparable with leading commercial sources of protein.

To evaluate the potential incorporation of thraustochytrids as an ingredient into the food industry, it is crucial to determine their functional properties. Important functional characteristics of proteins include their glass transition temperature, melting point, isoelectric point, molecular weight, secondary structure, solubility, surface hydrophobicity and emulsification [8]. These characteristics will establish their viability and best use in the food industry. The functional properties of thraustochytrid protein and oil are very limited. Thus, our study focusses on the characterization of thraustochytrid protein and oil and their suitability to be applied in the food industry.

Earlier, we showed that the oil isolated from thraustochytrid species could be used as a valuable source of docosahexaenoic acid (DHA), squalene, carotenoids and proteins [15]. In this study, we aimed to extract and characterize thraustochytrid protein isolate (TPI) from their oilcake to add value. We have determined the physicochemical properties of TPI for its potential use in the food and feed industries. To our knowledge, this is the first evaluation of the oilcake left after the extraction of oil from thraustochytrids and the first attempt to characterize TPI.

## 2. Materials and Methods

### 2.1. Growing Thraustochytrids

Two Australian strains of thraustochytrids, grouped into the genera *Aurantiochytrium* and designated as MAN65 and MAN70 (GenBank accession numbers MH790117, described in Nham Tran, Miranda [15]) were used. Both strains were grown using 3% (*w/v*) of glucose with 0.5% yeast extract and 1% tryptone in 50% seawater for seven days. The biomass was collected, washed in a sterile solution of 50% seawater and freeze-dried at −48 °C, 0.12 mbar for 48 h (Alpha 1–2 LD Plus Freeze Dryer and hybrid pump (Scitek, NSW, Sydney, Australia).

### 2.2. Preparatory Step for Extraction of Oil and Protein from Thraustochytrids

The freeze-dried thraustochytrid biomass was mixed with a mixture of methanol (meth) and chloroform at the former-to-latter ratio of 1:2. A solvent-to-biomass ratio of 1:6 was maintained and stirred for 3 h at room temperature. The mixture was then centrifuged at 3000 rpm at 20 °C for 15 min. The supernatant was collected for the extraction of oil, which was further purified (Section 2.2.1). The pellet was extracted three more times using a pellet-to-solvent ratio of 1:6 and stirring for 30 min at room temperature. The de-oiled thraustochytrid biomass (oilcake) was dried overnight in a fume hood.

#### 2.2.1. Oil Extraction

For oil extraction, 1% NaCl solution was added to the supernatant (Section 2.1) at an oil to-NaCl solution ratio of 1:6 which was mixed well and left to stabilize for 1 h. Then, the lower layer containing the oil was collected and dried in a rotary evaporator (R200 Rotovap, BÜCHI Labortechnik AG Flawil, Switzerland) at 40 °C, 85 torr and stored at −20 °C until analysis.

#### 2.2.2. Extraction of TPI

The oilcake was mixed with distilled water at a cake-to-water ratio of 1:20 (*w/w*). The mixture was aliquoted, and each aliquot was adjusted to a different pH. The chosen pH levels were 8.5, 10.5 and 12.0. The pH was adjusted with a 1 M NaOH or 1 M HCl solution and stirred for 2.5 h at two extraction temperatures (25 °C and 45 °C). The mixtures were centrifuged at 10,000 rpm at 4 °C for 20 min, and the supernatant was collected. To determine the optimal pH for precipitation of protein from the supernatant, nine different pH values ranging from 2.0 to 12.0 were used. The pH at which the electrostatic charge of the supernatant became neutral (zero zeta potential, or isoelectric point) was used to precipitate the protein. Once the protein was precipitated, the sample was centrifuged at 10,000 rpm for 20 min. The supernatant was discarded, and the pellet was collected and freeze-dried. The yield and the recovery of protein in the extracts were calculated using Equations (1) and (2), respectively.

(1) $$YE\;\left(\%\right)=\frac{protein\;extract\;\left(g\right)}{biomass\;\left(g\right)}\times 100$$

where YE is the yield of protein in percentage, protein extract is the mass/weight (in grams) of the pellet obtained after the final centrifugation of the precipitates, biomass is the mass/weight (in grams) of the oilcake used in the extraction process.

(2) $$Protein\;recovery\;\left(\%\right)=\frac{\;protein\;extract\;\left(g\right)\times protein\;in\;protein\;extract\;\left(\%\right)}{biomass\;\left(g\right)\times protein\;in\;biomass\;\left(\%\right)}\times 100$$

where protein in protein extract and protein in biomass are protein contents (in percentage) in the pellet and the oilcake, respectively as defined by Equation (1). Protein contents were measured using the Kjeldahl method as described below.

Considering the yield and total protein content of TPI extracted at different pH and temperatures, the final extraction was carried out at pH 12.0 and 25 °C.

### 2.3. Determination of Moisture, Lipid, Ash and Total Protein Contents of TPI

The moisture and ash contents in the TPI were determined using Association of Official Analytical Chemists (AOAC) standard methods 925.1 and 923.03, respectively [22]. Total lipid content was conducted following the method by Miranda, Liu [23]. The total protein content was measured using the Kjeldahl method (AOAC 991.22) by using a Kjeldahl Digestion System (KjeltecTM 8200, Foss, Hilleroed, Denmark). The nitrogen content of the samples was measured, and it was converted to protein content by multiplying by 6.25 [24].

### 2.4. Determination of Amino Acid Composition

The amino acid composition of the TPI was determined according to a commonly used standard method [25,26], except for tryptophan. Briefly, the TPI was hydrolyzed with 6 M HCl at 103 °C for 24 h. For tryptophan analysis, the hydrolysis was carried out using 5 M NaOH at 103 °C for 24 h. After hydrolysis, the hydrolyzed samples were analyzed by HPLC using an Agilent 1290 Infinity LC System [27] following the parameters 5990–5599EN of the Agilent method.

### 2.5. Determination of Protein Profile

Sodium dodecyl sulfate-polyacrylamide gel electrophoresis (SDS-PAGE) was used to qualitatively determine the protein profile in TPI according to the modified Laemmli method [28]. The TPI was mixed with Milli-Q water at pH 12.0 to a concentration of 5 mg mL^−1^, followed by centrifugation at 10,000 rpm at 4 °C for 1 min. The supernatant was then mixed with the loading buffer (95% Laemmli buffer and 5% 2-mercaptoethanol, *w/w*) using a sample-to-buffer ratio of 1:1 (*v/v*). This mixture was heated at 95 °C for 5 min. The test was carried out on a gel slab comprised of 5% stacking gel and 12% separating gel in an SDS–Tris–glycine discontinuous buffer system. A total of 12 µL of the sample was loaded in the specified lane, and the molecular size of the protein was determined by Invitrogen^TM^ molecular size standards. Electrophoresis was performed at 100 V for 90 min. The gels were finally stained with Coomassie brilliant blue R for 24 h.

### 2.6. Determination of Protein Solubility

TPI (100 mg) was dispersed in 10 mL Milli-Q water, and the pH of the mixture was adjusted from pH 2.0 to 12.0. The mixture was stirred for 2 h and allowed to hydrate overnight at 4 °C and then centrifuged at 18,000 rpm for 30 min at ambient temperature. The supernatant was collected, and protein content in the mixture was determined using a Bradford assay [29].

### 2.7. Measurement of Zeta Potential

TPI was mixed with Milli-Q water to form a 0.05% (*w/w*) solution, and then its pH was adjusted to 12.0, then 10.0; and then to 2.0 with 1 pH intervals. Then, a dynamic light scattering instrument (Zetasizer NanoZS, Malvern Instruments Ltd., Worcestershire, UK) was used to measure the zeta potential.

### 2.8. Determination of Surface Hydrophobicity

The surface hydrophobicity of the TPI was measured according to Kato and Nakai [30] with a minor modification. For this, 200 mg of TPI was mixed with 20 mL of a 20 mM phosphate buffer solution (pH 7.4) and the mixture was stirred for 3 h at 25 °C. The mixture was then centrifuged at 8000 rpm for 15 min at 4 °C. The collected supernatant was diluted with phosphate buffer using five supernatant-to-buffer ratios (1:2, 1:4, 1:6, 1:8, and 1:10). The protein concentration in these diluted samples was determined using a Bradford assay, as mentioned in Section 2.5. Twenty microliters of 8-anilino-1-naphthalene sulfonic acid (ANS) (8.0 mM in phosphate buffer, pH 7.0) was added to 4 mL of the diluted protein solution, and the fluorescence intensity (FI) of the protein was measured at excitation and emission wavelengths of 360 nm and 460 nm, respectively, using a spectrofluorometer (POLARstar Omega, BMG Labtech, Offenburg, Germany).

### 2.9. Determination of the Secondary Structure

A circular dichroism (CD) Spectropolarimeter 815 (Jasco International Co., Ltd., Tokyo, Japan) was used to observe the secondary structure of the TPI. The pre-weighed amount of TPI was mixed with sodium carbonate buffer at pH 9.2 to a final concentration of 1 mg/mL. Afterward, 8 µL of the sample was loaded into a cylindrical quartz cell with an optical pathlength of 0.1 mm with a wavelength range of 180–260 nm. Secondary structural features of the samples were estimated using the Dichroweb software, London, UK [31].

### 2.10. Determination of the Thermal Behavior of TPI

To determine the initial, mid-point (at 50% mass decomposition), and peak decomposition temperatures of TPI and thraustochytrid oil (TO), a thermogravimetric instrument was used. An accurately weighed amount of dried protein powder (7.34 mg) was placed in the pan and heated from 23 °C to 750 °C at a heating rate of 10 °C min^−1^ under a nitrogen flush.

To measure the denaturation temperature and denaturation enthalpy of TPI, the TPI sample was scanned using a differential scanning calorimeter (Q200, TA Instruments, New Castle, DE, USA). Protein powder (10 mg) was placed in an aluminum pan and hermetically sealed. A sealed empty pan was used as a reference. The sample was then heated from 25 °C to 180 °C at a rate of 10 °C min^−1^. The melting point of TO also was determined by using the same system and heating the sample from 20 °C to 60 °C at a rate of 10 °C min^−1^. All these tests were carried out in an inert environment using nitrogen.

### 2.11. Fourier Transform Infrared Spectroscopy (FTIR) Analysis

To characterize the chemical components in TPI and TO, the FTIR spectrum of the samples was determined in the wavenumber range of 400 to 4000 cm^−1^ at 4 cm^−1^ resolution using a MIRacel^TM^ ZnSe single reflection attenuated total reflection (ATR) (Perkin-Elmer, Norwalk, CT, USA). Thirty-two scans were acquired per sample.

### 2.12. Measurement of Emulsifying Properties

The emulsifying activity index (EAI) and stability (ESI) indices of TPI were measured according to Pearce and Kinsella [32] with a minor modification. TPI solution (10 mg/mL) was prepared by dissolving the protein at pH 8.0 at 40 °C for 40 min. Then, 2 mL of TO was added to 18 mL protein solution and homogenized at 13,500 rpm for 1 min using an ultra Turax (RW 20, IKA GmbH Co., Bitterfeld-Wolfen, Germany). The emulsion sample (50 μL) was pipetted from the bottom of the tube immediately after homogenization (0 min) and allowed to stand for 10 min. Then, 5 mL of 0.1% sodium dodecyl sulfate (SDS) solution was added into each emulsion and vortexed. The absorbance of the emulsions immediately after homogenization (A0) and standing for 10 min (A10) was measured at 500 nm using a UV-VS spectrophotometer (DR 5000™ Spectrophotometer, Hach, IA, USA). The EAI and ESI values were calculated using Equations (3) and (4), respectively.

(3) $$EAI\;\left(\frac{m^{2}}{g}\right)=\frac{2\times 2.303\times A0}{0.1\times WE}$$

(4) $$ESI=\frac{A0\times 10}{A_{0}-A_{10}}$$

where WE is the percentage of protein in mg/mL.

### 2.13. Statistical Analysis

All experiments were performed in triplicate, and the results are expressed as mean ± standard deviation (SD). Significant differences between two mean values were determined using the one-way analysis of variance (ANOVA) on the SAS 8.2 software platform. Differences were statistically significant at a 95% confidence level (*p* < 0.05).

## 3. Results and Discussion

### 3.1. Effect of Temperature and pH on the Yield, Recovery and Total Protein Content of TPI

**Figure 1:**
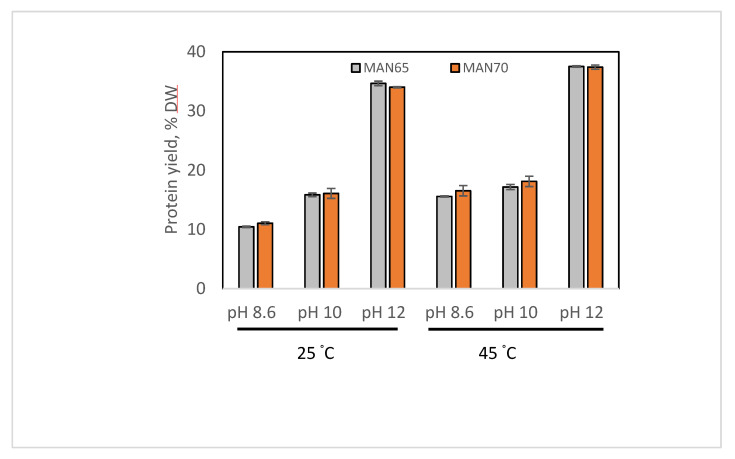
Yield of protein isolate extracted from two strains of thraustochytrids (MAN65 and MAN70) at different pH and temperature. DW: dry weight.

The schematic process flow diagram used to extract oil and TPI from cultured thraustochytrid cells is shown in Supplementary Figure S1. The yield of extracted TPI from both strains (MAN65 and MAN70) at different pH and the temperature is shown in Figure 1 and Table 1. For both strains, the yield of TPI was significantly higher at pH 12.0 than at the other two pH values. At both temperatures, the yield was increased up to 3.1- and 2.3-fold, respectively, at pH 12.0 compared to at pH 8.6. The increase of the protein yield at higher pH could be explained by the increase of protein solubility (Section 3.2) and increased separation from other compounds in thraustochytrid cells. At pH 10.0 and 12.0, the yield of TPI increased marginally when the temperature increased from 25 °C to 45 °C. However, the yield increased by 30% at 45 °C than at 25 °C. There was no significant difference in the yield of TPI extracted from the two strains (MAN65 and MAN70). The yield of protein from these two thraustochytrid strains was similar to those from other microalgae commonly used as sources of protein, with yields ranging between 15% to 74% at pH 12 depending on the species [33,34].

For both strains, the total protein content of TPI extracted at pH 12.0 and 45 °C decreased significantly (90% to 71%) compared to the one extracted at the same pH and 25 °C (Table 1). The lower purify of TPI extracted at a high temperature can be attributed to the increased extraction of extracellular polysaccharide substances (EPS). Thraustochytrids secrete EPS as part of their metabolism and remain attached to their cells [15,35]. A similar increase in protein yield was reported in other studies that evaluated the effects of pH and temperature during the alkaline extraction of proteins. Gerde, Wang [36] reported an increase of protein yield from about 2% at pH 9.0 (at 30 °C) to 30% at pH 13.0 (at 60 °C) from single-cell algae *Nannocloropsis* species. Kaushik, Dowling [9] reported a rise in the flaxseed protein yield from 4.5% at 25 °C to 5.36% at 50 °C at pH 8.6.

The data presented in Table 1 shows that the recovery of protein from these thraustochytrid strains is quite high (60–68%), which is remarkable as the recovery of protein from flaxseed and coconut at a similar pH and temperature range was reported to be 19% and 42%, respectively [37]. This high level of protein recovery is probably because unicellular thraustochytrid cells can be easily broken apart compared to plant cells.

### 3.2. Solubility and Surface Charge Density (Zeta Potential)

**Figure 2:**
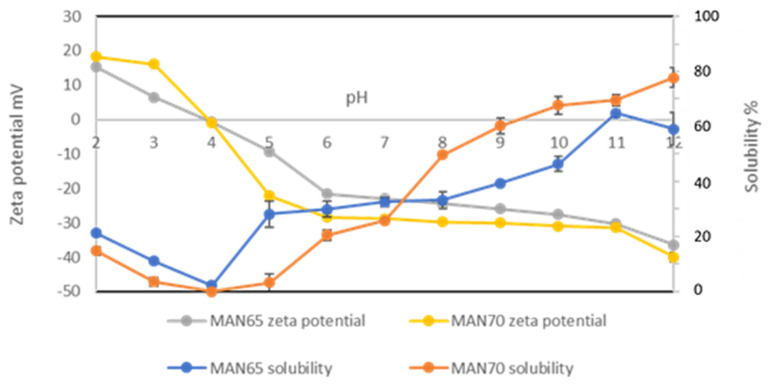
The zeta potential and solubility of thraustochytrid (MAN65 and MAN70) protein isolates as a function of pH.

The solubility of TPI as a function of pH is presented in Figure 2. The minimum solubility was recorded at pH 4.0, which is its isoelectric point, as shown by the zero zeta potential (charge neutral point) in TPI obtained from both strains. The solubility of TPI increased with increasing pH above pH 4.0 and reached the highest value at pH 12.0 (85% for MAN70 and 91% for MAN65).

The pH-dependent solubility profile of the TPI was similar to other protein isolates such as flaxseed and hemp [9,38]. The solubility of TPI from MAN65 was higher in acidic and neutral conditions (pH 4.0–7.0); however, the solubility of TPI from MAN70 at alkaline pH up to 11.0 was higher than that of MAN65. At the extraction pH (12.0), the solubility of TPI from both strains was similar to that of hemp protein [38] and slightly higher than flaxseed (80%) isolates [9]. However, at pH 7.0, the solubility of TPI was lower than that of flaxseed protein (57%) and similar to that of hemp protein (38%) isolates. This pH dependent-solubility of TPI with other oilseed proteins should be carefully considered in food processing because it provides an essential guide to the types of products proteins can be incorporated into [39].

The surface charge (zeta potential) as a function of pH is provided in Figure 2. The negative zeta potential of TPI at pH 12.0 was the highest, indicating the maximum exposure of anionic groups on the protein surface. The surface charge of TPI was reduced to zero at pH 4.0, indicating it to be an isoelectric point, which is corroborated by the lowest solubility at this pH.

### 3.3. Surface Hydrophobicity and Emulsifying Properties

The surface hydrophobicity of protein molecules relates to the extent of the hydrophobic amino acids that are exposed at the surface [40]. Surface hydrophobicity influences the intermolecular protein–protein and protein–lipid interactions, and it is an important property in the emulsion system because adsorption of protein at the oil–water interface is affected by hydrophobic interactions between the oil and hydrophobic protein patches [40]. The surface hydrophobicity of TPI extracted from MAN65 was 53.3 (dimensionless), which is significantly lower than that of MAN70 (60.85) (Table 2). This can be explained by the higher percentage of the amino acid alanine in strain MAN70 (Table 3). The hydrophobicity values of TPI were much lower than in oilseed protein isolates obtained from flaxseed (120) [41] and chia seed (100) [8].

The emulsifying activity index (EAI) and emulsion stability index (ESI) are important properties of proteins; the former indicates the interface area stabilized by per unit mass of the protein, while the latter indicates the ability of the emulsion to resist destabilization over a defined time [43]. The EAI values of TPI varied from 694.0 m^2^/g (MAN65) to 784.1 m^2^/g (MAN70) (Table 2), which is much higher than other sources of proteins such as flaxseed (160–350 m^2^/g) [9] and canola (25.1 m^2^/g) [44] or proteins such as bovine serum albumin (166 m^2^/g) or *k*-casein (185 m^2^/g) [45]. The ESI values of TPI ranged from 192.1 min (MAN65) to 209.9 min (MAN70) (Table 2). The higher ESI values in TPI compared to oilseed protein such as flaxseed (179.5 h) [9] indicate that it is a preferentially better emulsifier.

### 3.4. Thermal Characteristics of TPI

The thermal stability of a protein, indicated by its denaturation temperature (Td) and enthalpy of denaturation (∆H), is its ability to resist aggregation when heated [39]. The differential scanning colorimetry (DSC) heat flow versus temperature plot showed clear endothermic denaturation peaks at 167.8 °C for MAN65 and 174.5 °C for MAN70 (Table 2, Supplementary Figure S2). The higher thermal stability (higher Td) of TPI from MAN70 compared to that of MAN65 can be attributed to their different amino acid profiles. The Td of TPI obtained from both strains was higher than that of many other protein isolates such as from flaxseed (105 °C) [9], duckweed (103 °C) [46] and soybean (98 °C) [47]. Thus, the higher thermal stability of TPI from both strains indicates that they can be good ingredients as microencapsulating shell material and can be readily converted into powder form using thermally intense processes such as spray drying.

The thermal decomposition of TPI obtained from Thermo Gravimetric Analysis (TGA), indicating the initial decomposition temperature (IDT), temperatures at which 50% mass decomposition occurred (TD1/2), and maximum decomposition (MDT) occurred, are presented in Table 2. IDT values were observed at 233 °C (MAN65) and 242 °C (MAN70), both of which are lower than that of flaxseed protein isolate (259.3 °C) [41]. However, both the TD1/2 and MDT values of TPI are higher than that of flaxseed protein isolate. According to Pham, Wang [41], the TD1/2 and MDT of flaxseed protein isolate are 376 °C and 317.33 °C, respectively. These high decomposition temperature data also support that TPI is highly thermostable.

### 3.5. Approximate Amino Acid Composition of TPI

Table 2 presents the approximate composition of TPI extracted at 25 °C and pH 12.0. As can be observed, protein content in MAN70 is slightly higher than that in MAN65. However, the lipid and ash contents in MAN70 are marginally lower than that of MAN65, even though these differences are not significantly different.

The protein content in TPI (MAN65 and MAN70) was similar to that in flaxseed (~90%) [9], and chia seeds (~91%) [8] and higher than in other single-cell organisms such as algae where protein content is usually below 80% [33,48].

The amino acid composition of TPI from both strains is shown in Table 3, together with that of soy protein, Spirulina and flaxseed protein isolates for comparison. The amino acid composition of TPI from both strains was similar, except for threonine, arginine and alanine. The threonine (Thr) and arginine (Arg) contents in MAN65 (Thr = 10.4 mg/g, Arg = 150.8 mg/g) is significantly higher than that in MAN70 (Thr = 3.4 mg/g; Arg = 70.8 mg/g). The alanine content in TPI of MAN70 (49.4 mg/g TPI) is significantly higher than in MAN65 (10.8 mg/g TPI).

The essential amino acid composition of TPI obtained from both strains is similar to algae and plant protein isolates (Table 3), except for the fact that arginine and histidine contents are higher in the TPI compared to that in Spirulina, soybean and flaxseed. The high levels of arginine and histidine in TPI indicate that it is suitable for nutritional supplements. Histidine is a nutritionally essential amino acid that is also a precursor for several hormones (e.g., thyrotropin-releasing hormone), and critical metabolites affecting renal function, neurotransmission, gastric secretion, and the immune system [49,50,51]. When used together with L-carnosine, L-histidine has shown to impart an anti-aging effect in a neuronal cell line treated with D-galactose [52]. L-arginine is also shown to benefit the cardiovascular system [53] and improve muscle function [53].

The arginine/lysine (Arg/Lys) ratio has been shown to strongly positively affect the metabolic pathways of hypertension and have a positive effect on hypercholesterolemia, imparting lipidemic and atherogenic effects in rats even though the effects on humans were modest [54,55]. Even though the exact mechanism that leads to it positive effect is unknown, it was proposed that it is possible that this amino acid ratio has a role in cholesterol absorption by slowing the rate of lipid absorption, or it could lead to the elevation of the activity of the hepatic enzyme 7α-hydroxylase, which is a rate-limiting enzyme for the conversion of cholesterol to bile acids [56].

In this study, the Arg/Lys ratio is 3.16 for MAN65, which is similar to flaxseed (3.9) [9], and 1.09 for MAN70, which is a favorable ratio to be a useful protein ingredient in formulations intended to improve human health.

### 3.6. Molecular Weight of Main Fractions and Secondary Structure

The molecular weight of the main fractions of TPI determined by SDS-PAGE is shown in Supplementary Figure S3. As can be observed, TPI is a complex protein containing different molecular weight fractions (10 kDa to 100 kDa). The main bands in both strains were observed around 27 kDa and 37 kDa to 60 kDa. The blurred nature of these electrophoretic images is most probably due to some degree of denaturation of protein during extraction. The SDS-PAGE electrophoretic images show that the majority of these protein fractions are smaller than 50 kDa, as in the case of the single-cell organism algae *Tetraselmis* sp. [48]. It can also be seen that TPI also contains protein fractions that are larger than 50 kDa, as in the case of yeast protein [57].

**Figure 3:**
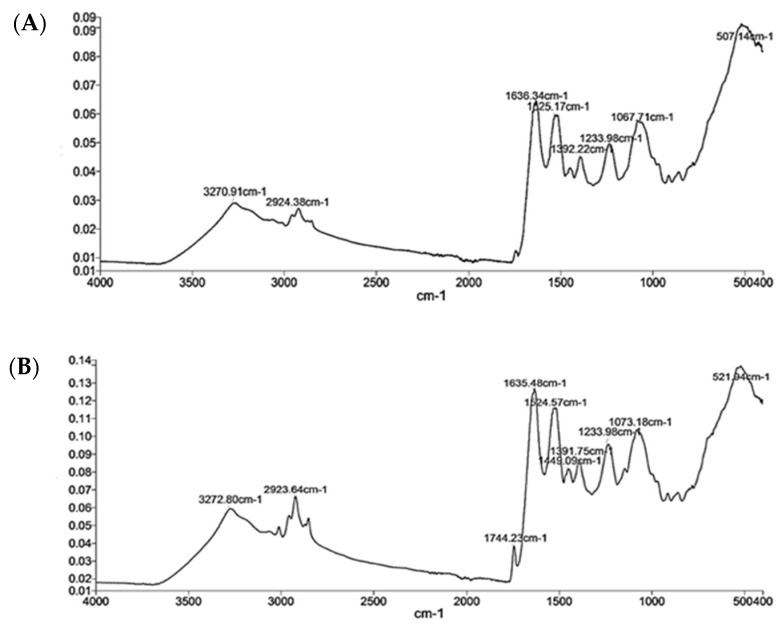
Fourier transform infrared (FTIR) spectra of thraustochytrid protein isolates obtained from two strains, MAN65 (**A**) and MAN70 (**B**).

The unique vibrations of structural units of a protein in FTIR spectroscopy reflect their secondary structure [58]. The protein repeats of thraustochytrid protein gave rise to five distinct IR absorption bands, namely amides A and I–II (Figure 3). The amide A band appeared at 3270 cm^−1^ and arose from N-H stretching. The amide I band appeared at 1636 cm^−1^, and it was the most sensitive spectral region associated with the protein’s secondary structure. In this band, the C=O vibrations have the predominant role, followed by C-N. There is also some in-plane N-H bending contribution to amide I [58,59,60].

The amide II band appeared at 1525 cm^−1^, and it had less sensitivity than the amide I band. The amide III band was a relatively weak and appeared at about 1300 cm^−1^. It was associated with N–H plane bending coupled with C–N stretching and C–H and N–H deformation vibrations. The absorption of IR energy in the 1200 cm^−1^ to the 900 cm^−1^ region is due to carbohydrate components present as contaminants [61].

The random coil structure of TPI (MAN65 and MAN70) was determined from the amide A and amide II bands that peaked around 3270 cm^−1^ and 1525 cm^−1^, respectively. The α-helix was determined from the intensity of the amide II band that peaked around 1525 cm^−1^. The β-sheet was determined by the intensity of the amide I band as indicated by its peak around 1636 cm^−1^ [62]. The secondary structure of TPI was primarily comprised of an α-helix, β-sheet, and random coil (Table 2). The random coil comprised 56 ± 1% of the secondary structure of both strains, indicating that the protein might have denatured, which was also shown in the blurred SDS-PAGE image. The α-helix content in TPI from strain MAN65 (10%) was lower than that in strain MAN70 (15%). The β-sheet content in TPI from strain MAN65 (34%) was higher than strain MAN70 (29%). The higher α-helix content in the TPI of MAN70 can be attributed to the fact that it contained higher concentrations of alanine, leucine and lysine, which prefer to adopt an α-helix structure. On the other hand, the tryptophan and threonine content is higher in TPI from MAN65, which generally prefer to adopt a β-sheet conformation [63].

### 3.7. Melting and Thermal Degradation of Thraustochytrid Oil

Earlier, we showed that thraustochytrid species MAN65 and MAN70 are rich in oil (up to 40% DW), which is rich in DHA, squalene, carotenoids and proteins [15]. Thermal stability of the oil is essential for processing and use and was measured in oils from both MAN65 and MAN70 using DSC (−20 °C to 60 °C) and TGA (30 °C to 600 °C). The thraustochytrid oils started to melt at about −7.5 °C, with their melting completed at 34.6 °C (MAN65 oil) at 30 °C (MAN70 oil) (Table 4).

**Figure 4:**
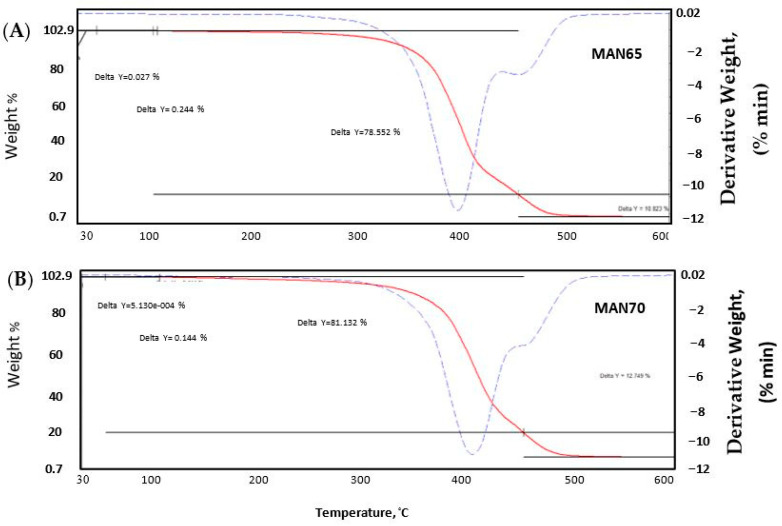
Thermogravimetric analysis (weight loss versus temperature and rate of weight loss versus temperature) plots of oils of thraustochytrids MAN65 (**A**) and MAN70 (**B**).

The mass loss versus temperature and derivative of mass loss versus temperature data of thraustochytrids obtained from TGA are presented in Table 4 and Figure 4. A small loss of oil mass (<1%, *w/w*) was observed when the oils were heated from room temperature to 213 °C in the case of MAN65, and 163 °C in the case of MAN70, indicating negligible degradation. The loss of mass increased rapidly above these two temperatures and reached a 50% mass loss when heated to 403 °C (MAN65) and 411 °C (MAN70). As can be observed from the derivative curve, the degradation of oil from both strains was more pronounced around 400 °C. Approximately 85% of the oil from both strains degraded when the temperature was increased to 470 °C (MAN65) and 463 °C (MAN70). The degradation continued upon further heating, and nearly all of the oil mass was volatilized when the temperature reached 500 °C.

### 3.8. Characteristic FTIR Spectra of Thraustochytrid Oil

**Figure 5:**
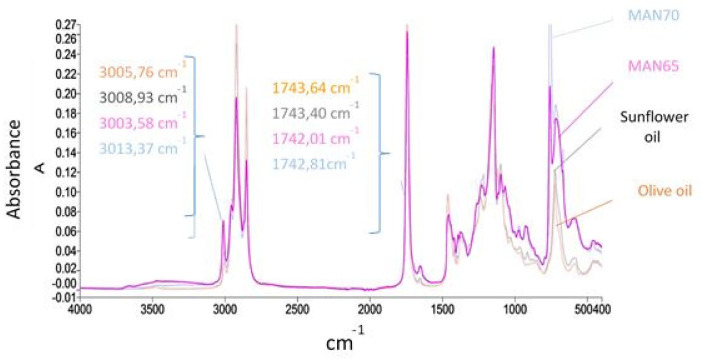
Comparison of Fourier transform infrared spectra of thraustochytrid oils (MAN65 and MAN70) with sunflower and olive oils.

Representative FTIR spectra of thraustochytrid oils (MAN65 and MAN70) and commercial olive and sunflower oils are shown in Figure 5. The characteristic spectral features of these oils were determined according to FTIR studies on PUFA-rich oils [64,65,66,67]. In thraustochytrid oil, the fist high-wavenumber region with strong triplet bands appeared in the 3020–2800 cm^−1^ range. The bands appearing in the 2950–2800 cm^−1^ range can be assigned to C–H stretching of the methyl and methylene backbones of lipids. The distinctive peak appearing around 3000 cm^−1^ indicates a stretching vibration of a cis double-bond of unsaturated fatty acids in most fish and marine oils due to their high concentration of DHA (C22:6) and EPA (C20:5) [64,67] The peak associated with this double bond appeared at 3005.8 cm^−1^ in olive oil, 3008.9 cm^−1^ in sunflower oil, and 3013.5 ± 0.1 cm^−1^ in thraustochytrid oil (MAN65 and MAN70). These observations indicate that MAN65 and MAN70 oil had a higher degree of unsaturation compared to sunflower oil and olive oil.

The second high-wavenumber region with prominent absorption bands appeared from 1742 cm^−1^ to 1744 cm^−1^, which indicates the C=O carbonyl stretching of the ester carbonyl functional group of the triglycerides of fatty acids [68,69]. This peak provides a measure of total lipid content in the analyzed oils. Thus, the ratio of area under the bands appearing around 3010 cm^−1^ and 1742 cm^−1^ is commonly used to determine the percentage of PUFA in the lipids. The ratio of the areas under the bands appearing at a frequency of 3010 cm^−1^ and 1742 cm^−1^ in thraustochytrid oil is three times higher than that in sunflower and olive oil, indicating that the PUFA content is much higher than that in sunflower and olive oil.

## 4. Conclusions

Protein isolate (TPI) and oil were extracted from two strains (MAN65 and MAN70) of thraustochytrids. The protein content in TPI was 90%, and it contained a high concentration of histidine and arginine. The Arg/Lys ratio in TPI was >1, which is considered to be healthy. The solubility of TPI in water was 30% at pH 7.0 and 85% at pH 12.0. TPI was found to be relatively heat stable as its denaturation temperature ranged from 167.80 °C for MAN65 and 174.50 °C for MAN70. Thraustochytrid oil melted at 30 °C (MAN70) and 34 °C (MAN65), and it was thermally stable up to 163 °C (MAN70) and 213 °C (MAN65). Given the potentiality of cultivating thraustochytrids and their oilcakes on a large scale, TPI can be produced at a much lower cost than plant and animal proteins. Given that alkaline extraction can potentially lead to chemical modification in proteins that can affect their solubility and other functional properties, extracting TPI in natural deep eutectic solvents followed by membrane filtration should be considered for realizing better nutritive and functional properties.

## Figures and Tables

**Table 1:** Extraction yield, recovery and total protein content of protein isolate from two strains of thraustochytrids (MAN65 and MAN70) extracted at two temperatures (25 °C and 45 °C) at pH 12.0.

Temperature (°C)	Strains	Protein Extraction Yield (%)	Protein Content (%)	Protein Recovery (%)
25	MAN65	34.6 ± 0.08	89.0 ± 0.7	66.9 ± 0.02
	MAN70	34.0 ± 0.04	91.6 ± 0.4	67.5 ± 0.02
45	MAN65	37.4 ± 0.01	70.5 ± 1.2	57.3 ± 0.02
	MAN70	37.5 ± 0.02	70.8 ± 0.5	54.9 ± 0.02

**Table 2:** The approximate composition, surface hydrophobicity, emulsion and thermal properties of thraustochytrid protein isolates (MAN65 and MAN70) extracted at pH 12.0 and 25 °C.

TPI Characteristics	MAN65	MAN70
Approximate Composition		
Moisture (%)	3.13 ± 0.85	4.30 ± 0.61
Protein (%)	91.64 ± 0.45	89.08 ± 0.79
Ash (%)	0.035 ± 0.12	3.65 ± 0.06
Lipid (%)	3.05 ± 0.07	3.13 ± 0.02
Surface hydrophobicity	53.33 ± 0.27	60.85 ± 0.93
Emulsion		
Emulsifying activity index (m^2^/g)	693.98 ± 2.83	784.12 ± 1.82
Emulsion stability index (min)	192.09 ± 1.75	209.86 ± 12.53
Secondary Structure		
Alpha helix (%)	10.00 ± 0.20	15.00 ± 0.10
Beta sheet (%)	34.00 ± 0.10	29.00 ± 0.20
Random coil (%)	55.00 ± 0.05	57.00 ± 0.05
Thermal Parameters		
Denaturation temperature (Td) (°C)	167.80 ± 0.50	174.50 ± 0.20
Denaturation enthalpy (ΔH) (°C)	3.33 ± 0.40	3.21 ± 0.40
Initial decomposition temperature (IDT) (°C)	233.00 ± 3.40	242.00 ± 1.60
Temperature 50 wt% decomposition (TD1/2) (°C)	232.96 ± 3.39	242.04 ± 1.56
Temperature of maximum of decomposition occurs (MDT) (°C)	342.40 ± 2.20	345.6 ± 0.30

**Table 3:** Amino acid composition of thraustochytrid (MAN65 and MAN70) protein isolates.

Amino Acid of TPI (mg/g)	MAN65 ^a^	MAN70 ^a^	SPI ^b^	SPN ^c^	FPI ^d^
Essential Amino Acids					
Aspartic acid	72.4	90.7	118	60.5	101.8
Alanine	10.8	49.4	38.3	117.5	43.6
Arginine	158.2	70.8	75.7	3.6	108
Glutamic acid	146.5	143.7	212.9	112.3	185.1
Glycine	16.1	19	38.6	72.3	48.2
Histidine	110.6	132.6	29	9.2	21.8
Serine	33.7	40.5	54.8	26.9	47
Threonine	10.5	3.4	41	25.6	33.9
Tyrosine	22.6	26.1	37.1	9.8	25.6
Non-Essential Amino Acids					
Cysteine	5.6	4.3	0.6	51.5	10.7
Isoleucine	22.9	30.2	44.8	15.7	45.4
Leucine	47.6	61.3	70	18.1	54.9
Lysine	50	64.7	53.9	36.4	27.5
Methionine	14.2	13.8	9.3	45.2	18.6
Phenylalanine	27.1	35.1	53	10.1	53.1
Proline	33.9	34.5	52.9	7	37.7
Tryptophan	41.9	36.6	nr	7.5	20.4
Valine	34.4	43.4	44.1	41.7	55.2

Comparative values of soy protein (SPI), Spirulina (SPN), and flaxseed protein (FPI) isolate. ^a^ Data from this study, ^b^ [38], ^c^ [42], ^d^ [9].

**Table 4:** The thermal characteristics of oil from thraustochytrids (MAN65 and MAN70).

Characteristics	MAN65	MAN70
First melting point (°C)	−7.8 ± 0.0	−7.6 ± 0.4
Second melting point (°C)	34.6 ± 0.6	29.2 ± 0.3
Temperature at 1 wt% decomposition (°C)	213.3 ± 8.2	163 ± 1.7
Temperature at 50 wt% decomposition (°C)	403.5 ± 0.8	410.9 ± 0.4
Temperature at >85 wt% decomposition (°C)	469.7 ± 1.9	462.4 ± 4.4

## Supplementary Materials

The following are available online at https://www.mdpi.com/2304-8158/9/6/779/s1, Supplementary Figure S1: Schematic process flow diagram used to extract oil and TPI from cultured thraustochytrid cells., Supplementary Figure S2: Differential scanning calorimetry of TPI. (**A**) MAN65 and (**B**) MAN70., Supplementary Figure S3: SDS-PAGE electrophoresis of thraustochytrid protein isolates obtained from its two strains, MAN65 (**A**) and MAN70 (**B**).
