# Supplementary material for: Physicochemical Characteristics of Protein Isolated from Thraustochytrid Oilcake
Source: Foods. 2020 Jun 11;9(6):779. doi: 10.3390/foods9060779 (PMC7353575; doi:10.3390/foods9060779)
Supplement: Supplementary file 1 [file foods-09-00779-s001.pdf]

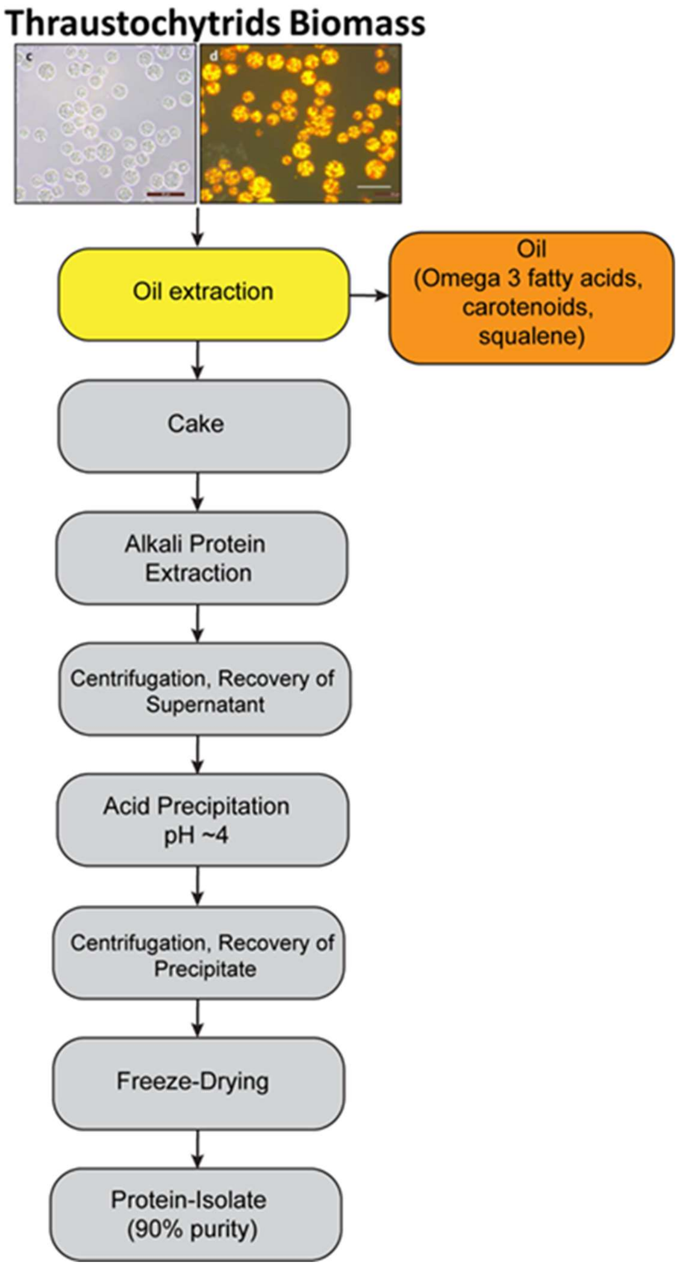

Figure S1. Schematic process flow diagram used to extract oil and TPI from cultured thraustochytrids cells**.**

(**A**)

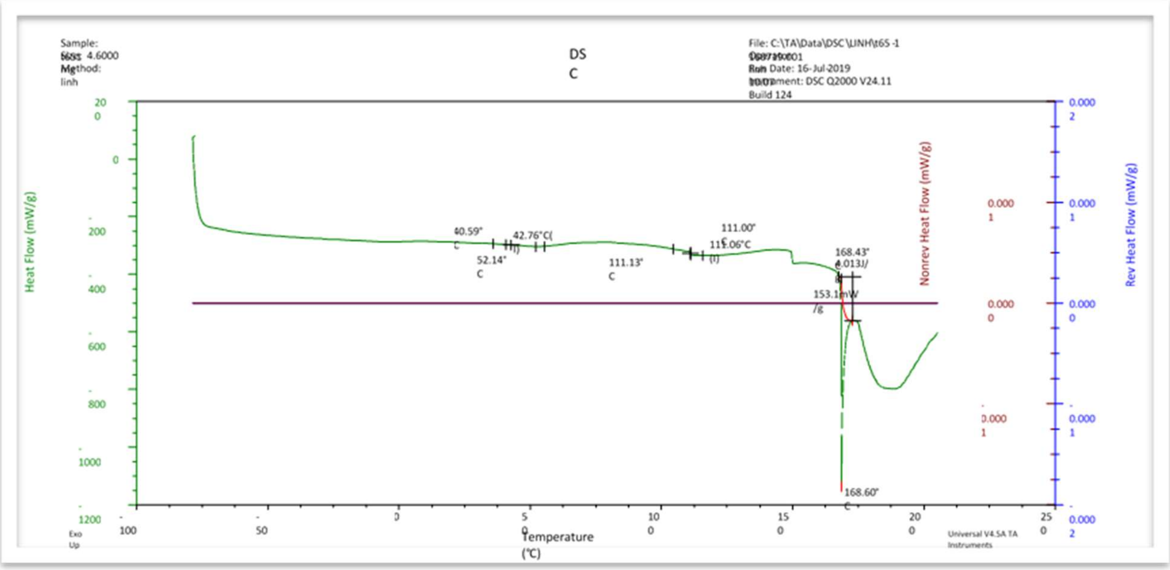

(**B**)

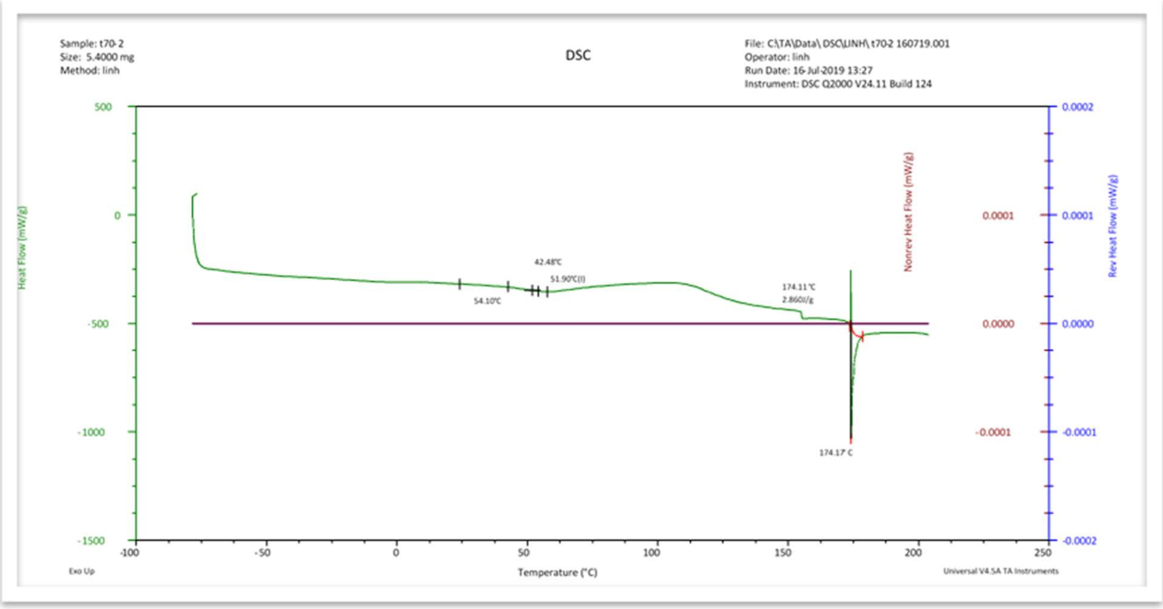

Figure S2. Differential scanning calorimetry (DSC) of TPI. (**A**) MAN65; (**B**) MAN70.

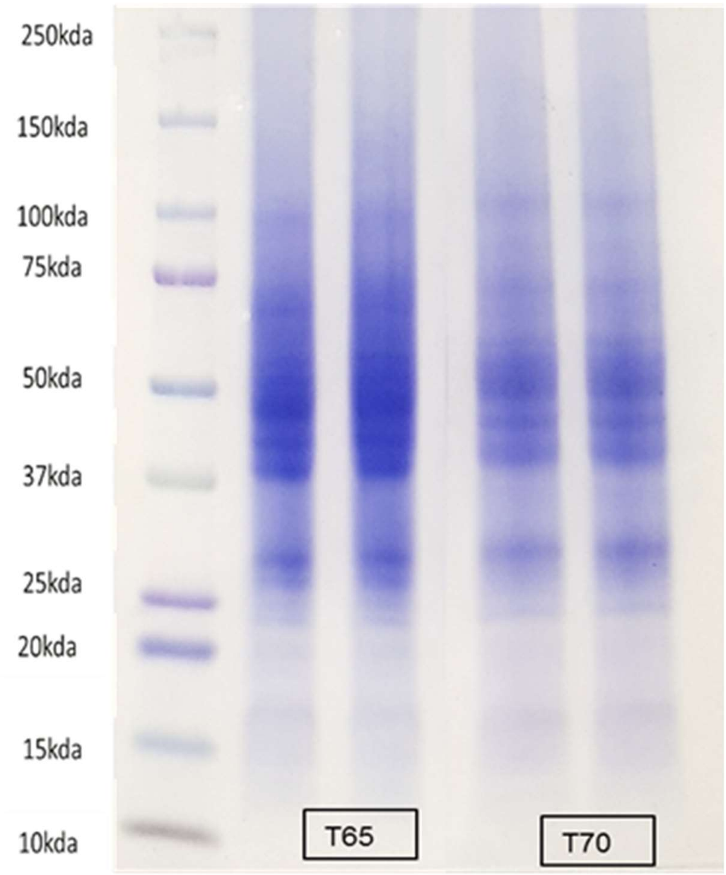

Figure S3. SDS-PAGE electrophoresis of thraustochytrids protein isolate obtained from its two strains MAN65 (**A**) and MAN70 (**B**).
